# Effects of 1,25 and 24,25 Vitamin D on Corneal Epithelial Proliferation, Migration and Vitamin D Metabolizing and Catabolizing Enzymes

**DOI:** 10.1038/s41598-017-16698-3

**Published:** 2017-12-05

**Authors:** Xiaowen Lu, Zhong Chen, Namratha Mylarapu, Mitchell A. Watsky

**Affiliations:** 1 Department of Cellular Biology and Anatomy, Medical College of Georgia at Augusta University, Augusta, Georgia USA; 2 The Graduate School, Augusta University, Augusta, Georgia USA

## Abstract

This study investigated the effects of 1,25(OH)_2_D3 and 24R,25(OH)_2_D3 on corneal epithelial cell proliferation, migration, and on the vitamin D activating enzyme CYP27B1 (produces 1,25(OH)_2_D3) and inactivating enzyme CYP24A1 (produces 24R,25(OH)_2_D3). The role of the vitamin D receptor (VDR) was also examined. In VDR wildtype mouse corneal epithelial cells (WT), 1,25(OH)_2_D3 increased CYP24A1 protein expression and decreased CYP27B1 expression. In VDR knockout mouse epithelial cells (KO), 1,25(OH)_2_D3 increased CYP24A1 and CYP27B1 protein expression. 1,25(OH)_2_D3 did not affect WT cell proliferation, but did stimulate VDR KO cell proliferation. In a human corneal epithelial cell line (HCEC), 1,25(OH)_2_D3 increased CYP24A1 mRNA and protein expression. 1,25(OH)_2_D3 increased CYP27B1 mRNA levels in HCEC, but had no effect on CYP27B1 protein levels. 1,25(OH)_2_D3 inhibited HCEC proliferation and stimulated cell migration in primary human epithelial cells. 24,25(OH)_2_D3, on the other hand, increased both CYP24A1 and CYP27B1 protein expression in WT and VDR KO cells, and stimulated cell proliferation in both WT and KO cells. In HCEC, 24,25(OH)_2_D3 increased CYP24A1 and CYP27B1 mRNA and protein expression, and stimulated cell migration. In human primary corneal epithelial cells, 24,25(OH)_2_D3 stimulated migration. We conclude that 24R,25(OH)_2_D3 is likely involved in corneal epithelial cell regulation independent of 1,25(OH)_2_D3 or VDR.

## Introduction

The vitamin D3 endocrine system is fundamental to health maintenance and disease prevention. Epidemiologic research has demonstrated that a low vitamin D concentration increases the risk for diseases including autoimmune and cardiovascular diseases, cancer, and metabolic disorders^1^. The classical systemic route for activation of vitamin D3 is initial 25-hydroxylation to 25-dihydroxyvitamin D3 (25(OH)D3; See Table 1 for list of all abbreviations) in the liver via cytochrome P-450 containing enzymes, followed by renal conversion to the active 1,25-dihydroxyvitamin D3 (1,25(OH)_2_D3) by 1α-hydroxylase (CYP27B1). 25(OH)D3 can also be hydroxylated by 25-hydroxyvitamin D-24-hydroxylase (CYP24A1) to produce 24R,25-dihydroxyvitamin D3 (24R,25(OH)_2_D3). Accordingly, CYP27B1 and CYP24A1 are integral to vitamin D metabolism^2,3^. Both 24R,25(OH)_2_D3 and 1,25(OH)_2_D3 undergo additional catabolism via multiple side chain hydroxylations to become more polar metabolites which are subsequently excreted in both the urine and the feces. These final vitamin D catabolism steps are carried out by multiple enzymes^4,5^.

**Table 1:** Abbreviations.

Vitamin D3 receptor	VDR
1,25-dihydroxyvitamin D3	1,25(OH)_2_D3
24R,25-dihydroxyvitamin D	24R,25(OH)_2_D3
1α-hydroxylase	CYP27B1
25-hydroxyvitamin D-24-hydroxylase	CYP24A1
3-(4,5-dimethylthiazol-2-yl)-2,5-diphenyltetrazolium bromide	MTT
Wildtype	WT
Heterozygous	HET
Knockout	KO
Human corneal epithelial cell line	HCEC
Human primary corneal epithelial cell line	HPCEC
VDR knockout mouse epithelial cells	VDR KO MPCEC
VDR wildtype mouse corneal epithelial cells	VDR WT MPCEC
Keratin 12	K12
TATA box binding protein	TBP

The kidney has long been viewed as the primary site expressing CYP27B1. CYP27B1 expression has also been reported in various tissues including skin, lymph nodes, hair follicles, colon epithelium, islets of the pancreas, and adrenal gland^6^. Our lab has demonstrated that VDR and 1α-hydroxylase are present in the eye, and that 25(OH)D3 and 1,25(OH)_2_D3 can both enhance the barrier function of corneal epithelial cells^7^. CYP24A1 has been found in target cells from several tissues including kidney and intestine, skin, thymus, bone, lung, testis, spleen, pancreas, heart, and recently in cultured corneal epithelial cells^8,9^. Few published clinical or basic science studies have examined the regulation of CYP24A1 and CYP27B1 or the physiological roles of vitamin D3 in the anterior segment of the eye^7,9^, particularly as they pertain to 24R,25(OH)_2_D3.

1,25(OH)_2_D3 is considered to be the most biologically active vitamin D metabolite. The classical nuclear vitamin D receptor (VDR) is considered the primary receptor for 1,25(OH)_2_D3. Membrane-associated protein disulfide isomerase family A member 3 (Pdia3) has been shown to be a secondary receptor for 1,25(OH)_2_D3, with each receptor separately activating its own downstream mediators^10,11^. Pdia3 binding of 1,25(OH)_2_D3 has been shown to control extracellular Ca^2+^ homeostasis and regulate bone growth and metabolism^11–13^. 1,25(OH)_2_D3 binding with VDR was first found to promote anti-proliferative and pro-differentiation responses in cancer cells^14–16^. Since then, many groups have reported similar anti-proliferative effects of 1,25(OH)_2_D3 and VDR *in vitro* and *in vivo* ^17^. Another important pronounced effect of 1,25(OH)_2_D3 is promotion of increased CYP24A1 synthesis, accelerating the catabolism of 1,25(OH)_2_D3^18^. Immunomodulatory activity of 1,25(OH)_2_D_3_ was recently demonstrated in corneal epithelial cells where it was shown to attenuate proinflammatory mediators while increasing antimicrobial peptides and antipseudomonas activity. In this same study, the expression of CYP24A1 was found to be increased and regulated by VDR in corneal epithelial cells cultured with 1,25(OH)_2_D3^9^.

24R,25(OH)_2_D3 is the most abundant dihydroxylated vitamin D3 metabolite^19,20^. 24,25(OH)2D3 generally circulates at concentrations of 1–10 ng/mL (2–20 nM), which is close to 100-fold higher than 1,25(OH)2D3^21^. In our lab, 60 nM 24,25(OH)2D3 and 0.2 nM 1,25(OH)2D3 was detected in rabbit plasma^22^. 24R,25(OH)_2_D3 is typically considered a waste product, although several studies indicate that it is indeed an active metabolite. Forty years ago, it was reported that 24R,25(OH)_2_D3 stimulated intestinal calcium uptake and aided bone repair without causing bone calcium mobilization^23^. A more recent study shows it may have a direct effect on bone formation^24^. 24R,25(OH)_2_D3 has also been shown to inhibit proliferation and stimulate osteocalcin expression in human (MG63) osteoblasts^25^. In addition, 24R,25(OH)_2_D3 was found to strongly increase CYP24A1 expression in primary human osteoblasts^25^. 24R,25(OH)_2_D3 has also been found to promote differentiation of human mesenchymanl stem cells^24^. No studies have examined the influence of 24R,25(OH)_2_D3 on the cornea or corneal epithelium.

The aims of this study were to investigate the effects of 1,25(OH)_2_D3 and 24R,25(OH)_2_D3 on corneal epithelial cell proliferation, migration, and on the expression of the vitamin D activating enzyme CYP27B1 and the vitamin D metabolizing enzyme CYP24A1 in human and mouse corneal epithelial cells. VDR knockdown and knockout corneal epithelial cells were utilized to examine the role of the VDR in these responses.

## Methods

### Materials

1,25(OH)2D3 and 24R,25(OH)2D3 were purchased from Sigma (Ann Arbor, MI). CYP24A1 and CYP27B1 polyclonal antibodies were purchased from Santa Cruz Biotechnology (Santa Cruz, CA). VDR and GAPDH antibodies were purchased from Cell Signaling Technology (Danvers, MA). Prestained protein markers were obtained from Bio-Rad (Hercules, CA). Polyvinylidene difluoride (PVDF) membrane and the enhanced chemiluminescence (ECL) detection system were obtained from Bio-Rad (Hercules, California).

### Human Corneal Epithelial Cell Line and Primary Epithelial Cells

The immortalized human corneal epithelial cell line (HCEC) utilized in this study has been previously described^7,26,27^. HCEC were grown on standard culture plates until confluent. All cells were grown in Dulbecco’s modified Eagle’s medium (Invitrogen, Carlsbad, CA) supplemented with FBS (3%), 1% ITS (BD Biosciences, Bedford, MA), and 40 μg/mL gentamicin (Life technologies, NY). The cells were subpassaged using trypsin (Sigma, Ann Arbor, MI) digestion, seeded in 35 mm dishes (Fisher Scientific, PA), and cultured in a humidified incubator at 37 °C with 5% CO_2_. The culture medium was replaced with fresh DMEM medium plus 3% serum every 2 days.

Primary human epithelial cells were obtained from de-identified donor corneal rims courtesy of Dr. Amy Estes from the Department of Ophthalmolgy, Medical College of Georgia, Augusta University. Corneas from 3 donors were used, and all cells were obtained from transplant corneal rims within 24 hours after the surgeries were performed. The donors were from: 32 year old male, 33 year old male, and 58 year old male. Primary human epithelial cells were cultured using the same methods as for HCEC. Cells from passage three to six were used for experiments. The presence of keratin 12 and mucin 1 mRNA was tested by PCR to confirm immortalized and primary cell types (data shown in Supplemental Figure S1).

### VDR silencing

HCEC cells were transiently transfected with siRNA using Lipofectamine 2000 reagent (Invitrogen, Carlsbad, CA) according to the manufacturer’s specifications based on established protocols using 100 nmol VDR siRNA (sc-106692, Santa Cruz Biotechnology, Dallas, TX), or a control siRNA (sc-37007, Santa Cruz Biotechnology) for 48 hours. VDR-silenced cells were treated with 1,25(OH)_2_D3 (10 nM) or 24R,25(OH)_2_D3 (100 nM) for 18 hours.

### Mouse primary corneal epithelial cells (MPCEC)

Wildtype (WT), heterozygous (HET) and VDR knockout (KO) mice were obtained and bred from the Jackson Labs (Strain: B6.129S4-Vdr^tm1Mbd^). All animal studies were approved by the Augusta University IACUC, and methods were performed and animals treated in accordance with the Augusta University IACUC guidelines and regulations as well as the ARVO statement for the Use of Animals in Ophthalmic and Visual Research. Murine cells gave us the opportunity to study the effects of full VDR knockout, as opposed to only siRNA knockdown, on vitamin D signaling in corneal epithelium. Primary mouse corneal epithelial cell cultures were established using a modification of an established explant culture method^28,29^. Many VDR knockout mice die before 12 weeks of age, and the phenotype is usually expressed between 4 and 5 weeks of age (rickets type symptoms, alopecia, etc,), thus we collected corneas from mice as early as 4 weeks of age. Briefly, 4-week-old mice were killed, the eyes were enucleated, and cornea buttons were obtained using a pathogen-free disposable punch (2.5 mm) and washed with Ca^2+^ free PBS (pH 7.2). Each cornea was cut in half and placed in a 35 mm dish (Fisher Scientific, PA) with the epithelial side up. After approximately 5 minutes in the laminar air-flow culture hood, the half corneal button attached itself to the bottom of the plate, and 1.5 mL of DMEM (Life technology, NY) with 10% serum containing 40 μg/mL gentamicin (Life technologies, NY), 1% ITS (BD Biosciences, Bedford, MA), and 100 ng/mL cholera toxin (LIST Biological Laboratories, Inc., Campbell, CA) were added and the tissue was placed in a humidified incubator at 37 °C with 5% CO_2_. Culture medium was replaced every 2 days. After 7 to 10 days cells were nearly 100% confluent.

Cells were passaged using 2.5% trypsin (Sigma, Ann Arbor, MI) with gentle pipetting, centrifuged at 500 g for 5 minutes, and subcultured in DMEM with 3% serum containing 40 μg/mL gentamicin, 1% ITS, and 100 ng/mL cholera toxin.

### Cell Migration Assay

Migration assays were performed using a scratch-wound protocol. Primary human corneal epithelial cells were seeded onto 35 mm cell culture dishes (3.5 × 10^4^ cells/dish), incubated at 37 °C until they attained 80–90% confluence, and then starved in DMEM plus 0.1% fetal bovine serum overnight at 37 °C. Cells were scratch-wounded using a sterile 10 μL pipette tip, washed two to three times with medium to remove all loose or dead cells, photographed (0 hours), and 1,25(OH)_2_D3 (10 nM) or 24R,25(OH)_2_D3 (100 nM) was added. To avoid cell proliferation in the scratch assay, serum free medium was used for the assay, and the assay was limited to 18 hours duration. Control dishes were similarly scratch-wounded and incubated without addition of vitamin D metabolites. Cells that had migrated across marked reference lines were photographed using a Hoffman contrast-equipped microscope (Olympus, Tokyo, Japan). The extent of healing over time was defined as the ratio of the difference between the original wound area and the remaining wound area after 18 hours. Data was analyzed from 10 scratches, using cells from 3 independent donors. The area of migrating cells was analyzed using CellSens Dimension software (Olympus, Tokyo, Japan).

### Cell Proliferation

HCEC and MPCEC were seeded onto 24-well cell culture plates (5 × 10^4^ cells/dish), incubated at 37 °C until plates reached 55–65% confluence, and then starved in DMEM plus 0.1% fetal bovine serum overnight at 37 °C. Cells were stimulated with 1,25(OH)_2_D3 (10 nM) or 24R,25(OH)_2_D3 (100 nM). The MTT [3-(4,5-dimethylthiazol-2-yl)-2,5-diphenyltetrazolium bromide] reduction assay was used as an index of viable cell density and was performed according to the manufacturer’s instructions (Fisher Scientific, PA). Control and vitamin D-treated cells were incubated in serum-free medium containing 0.4 mg/mL MTT. During this time, mitochondrial and cytosolic dehydrogenases of living cells reduced the yellow tetrazolium salt (MTT) to a purple formazan dye capable of spectrophotometric detection. After 2 to 2.5 hours, the MTT solution was aspirated and dimethylsulfoxide (0.3 mL/well) was added. Optical densities of the supernatant were read at 540 nm using a microplate spectrophotometer (BioTek, Winooski, VT). Absorbances were normalized to the control cultures, which represented 100% viability. Growth ratios were calculated as the ratio of the absorbance of vitamin D-treated cells versus control cells.

### Real Time PCR

Total RNA was obtained from HCEC, primary mouse epithelial cells, and primary human epithelial cells. Real time PCR was used to quantify CYP24A1 and CYP27B1 mRNA levels. GAPDH was used as the internal RNA control for human corneal epithelial cells, and TATA box binding protein (TBP) was used as the mouse corneal epithelial cell RNA control. The RT-PCR primers for human and mouse CYP24A1, mouse CYP27B1, human GAPDH, and human VDR were generated from the PrimerBank database^30,31^ using National Center for Biothechnology Information (NCBI) sequence identification numbers (NM_009996, NM_010009, NM_001017536, NM_001289746.1, NM_001128915 for mouse CYP24A1, mouse CYP27B1, human VDR, human GAPDH, and human CYP24A1, respectively). The primers for CYP27B1 were designed using data from human decidual cells^2,32^. The primers for mouse TATA box binding protein (TBP) were generated from Universal ProbeLibrary of Roche Life Science. The primer specificity was validated with melting profiles and the electrophoresis gels of RT-PCR products (data shown in supplementary Figures S2–S4). Primers are listed in Table 2.

**Table 2:** Summary of primers pair sets.

Gene	Direction	Primer Sequence	Product Size	Melting Temperature
Mus musculus CYP24A1^a^	Forward	5′-CTGCCCCATTGACAAAAGGC-3′	148	61 °C
	Reverse	5′-CTCACCGTCGGTCATCAGC-3′		
Mus musculus CYP27B1^a^	Forward	5′-GGGCCAATATGGTCTGGCAG-3′	98	60 °C
	Reverse	5′-GGACAGTGACTTTCTTGTCGC-3′		
Mus musculus TBP^a^	Forward	5′- GGCGGTTTGGCTAGGTTT-3′	83	59 °C
	Reverse	5′-GGGTTATCTTCACACACCATGA-3′		
Mus musculus K12	Forward	5′-CATGGCTGAGCAAAATCGGAA-3′	187	61 °C
	Reverse	5′-CAGGGACGACTTCATGGCG-3′		
Homo sapiens CYP24A1^a^	Forward	5′-GATTTTCCGCATGAAGTTGGGT-3′	122	60 °C
	Reverse	5′-CCTTCCACGGTTTGATCTCCA-3′		
Homo sapiens CYP27B1^a^	Forward	5′-CACCCGACACGGAGACCTT-3′	60	61 °C
	Reverse	5′-TCAACAGCGTGGACACAAACA-3′		
Homo sapiens VDR	Forward	5′-GACTTTGACCGGAACGTGCCC-3′	228	62 °C
	Reverse	5′-CATCATGCCGATGTCCACACA-3′		
Homo sapiens GAPDH^a^	Forward	5′-CTGCACCACCAACTGCTTAG-3′	120	60 °C
	Reverse	5′- GGGCCATCCACAGTCTTC T-3′		
Homo sapiens K12	Forward	5′-TTCCATGTTTGGTTCTAGTTCCG-3′	171	59 °C
	Reverse	5′-TCATTGCCCGAGAGAATACCTA-3′		
Homo sapiens Mucin 1	Forward	5′-CCTGCCTGAATCTGTTCTGC-3′	126	60 °C
	Reverse	5′-CATGACCAGAACCCGTAACA-3′		

^a^The efficiency of CYP24A1, CYP27B1, GAPDH and TBP RT-PCR primers were measured using Cq slope method (data shown in Supplemental Table S1).

mRNA was isolated and cDNA was synthesized using the Bio-Rad RT-PCR system. First-strand synthesis was done at 42 °C for 60 minutes, and inactivated at 85 °C for 5 minutes. Equal amounts of cDNA were applied for PCR amplification in triplicate using the Bio-Rad system and SYBR probes. Amplification was performed at 95 °C for 10 minutes, followed by 40 cycles at 95 °C for 10 seconds and 60 °C for 30 seconds. Quantitative values were obtained from the quantification cycle (C_q_). Each sample was normalized on the basis of its gene content (∆C_q_). The formula 2^−(∆∆Cq)^ was used to analyze the results. Bio-Rad CFX Manager 3.1 software was used for RT-PCR data analysis

### Protein Extraction and Western Blot Analysis

Protein was isolated from confluent cells grown on 35 mm dishes by washing in PBS at 4 °C and adding lysis buffer (50 mM Tris-HCl [pH 8], 150 mM NaCl, 0.02% N3Na, 100 μg/mL phenylmethylsulfonyl fluoride, 1% NP-40, 50 mM NaF, 2 mM EDTA, and protease inhibitor [Sigma, Ann Arbor, MI]). Cell lysates were collected and Western blotting was performed as previously described^7^. Blots were labeled with CYP24A1 or CYP27B1 antibody at a dilution of 1:1000. Membranes were washed and then incubated with anti–rabbit horseradish peroxidase-conjugated secondary antibody (1:3000). Detection was performed using the enhanced chemiluminescence method (Pierce Biotechnology, Rockford, IL). To ensure equal protein loading, nitrocellulose membranes were labeled with GAPDH antibody at a dilution of 1:6000 (Cell Signaling Technology, Danvers, MA). Both X-ray film and the ChemiDoc™ XRS^+^ Imaging Systems (BIO-RAD, Hercules, CA) were used for chemiluminescence detection. For ChemiDoc™ blots, stain-free gels were used. While GAPDH was always run on the same gel as the experimental protein, GAPDH required substantially lower exposure times on the stain-free gels, thus the GAPDH section of the gels were cut out from the full gel and imaged separately. For this reason, all stain-free gel results show separated GAPDH sections.

### Statistical Analysis

All data are given as the mean ± SD of at least three experiments. Where applicable, differences between two groups were compared using the unpaired Student’s *t*-test. For multigroup comparisons, ANOVA followed by Student-Newman-Keuls test were performed. *P* < 0.05 was considered statistically significant.

### Data Availability

The datasets generated and/or analyzed during the current study are available from the corresponding author on reasonable request.

## Results

### Migration

**Figure 1:**
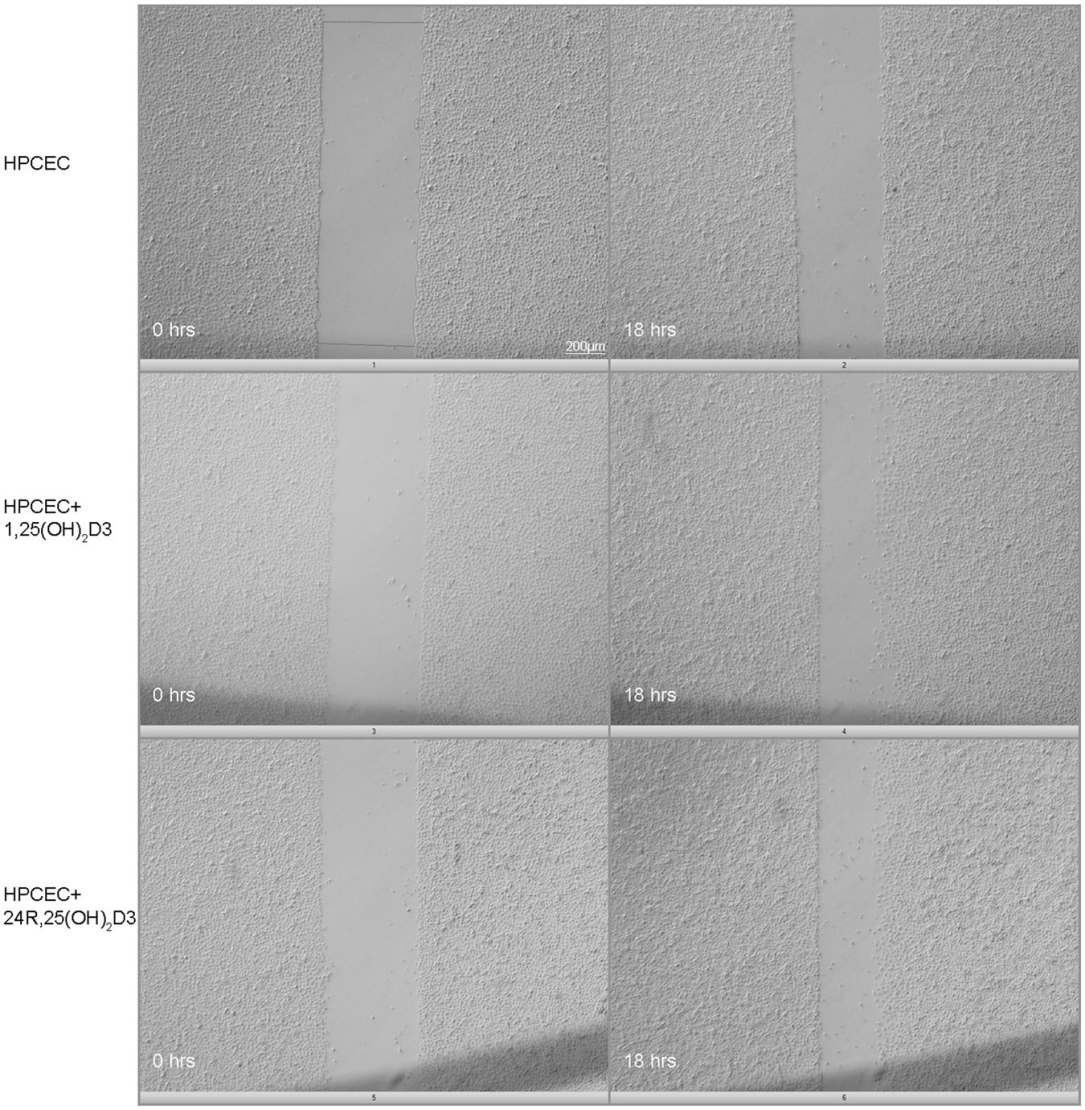
Human primary corneal epithelial cells were treated with 10 nM 1,25(OH)_2_D3 or 100 nM 24R,25(OH)_2_D3 for 18 hrs, and cell migration was assessed by the cell scrape assay. Representative pictures of control, 1,25(OH)_2_D3, and 24R,25(OH)_2_D3 (n = 10 scratches from 3 donors) are shown. Both 1,25(OH)_2_D3, and 24R,25(OH)_2_D3 significantly increased cell migration (Table 3).

1,25(OH)_2_D3 (10 nM) and 24R,25(OH)_2_D3 (100 nM) significantly increased HPCEC cell migration after 18 hours of treatment. (Fig. 1 and Table 3).

**Table 3:** HPCEC were treated with 10 nM 1,25(OH)2D3 or 100 nM 24R,25(OH)2D3 for 18 hrs.

Treatment	HPCEC		
Area	Control	1,25(OH)_2_D3	24R,25(OH)_2_D3
Mean	3194.9	4830.9*	5468.6*
SD	956.2	1392.3	1653.2

Cell migration was assessed by the cell scrape assay. Degree of cell migration was monitored by quantifying the wound area at 0 and 18 hours. Migration was significantly enhanced in 24R,25(OH)2D3 treated and 1,25(OH)2D3 treated HPCEC (ANOVA, P < 0.05, n = 10) versus control.

### Proliferation

**Figure 2:**
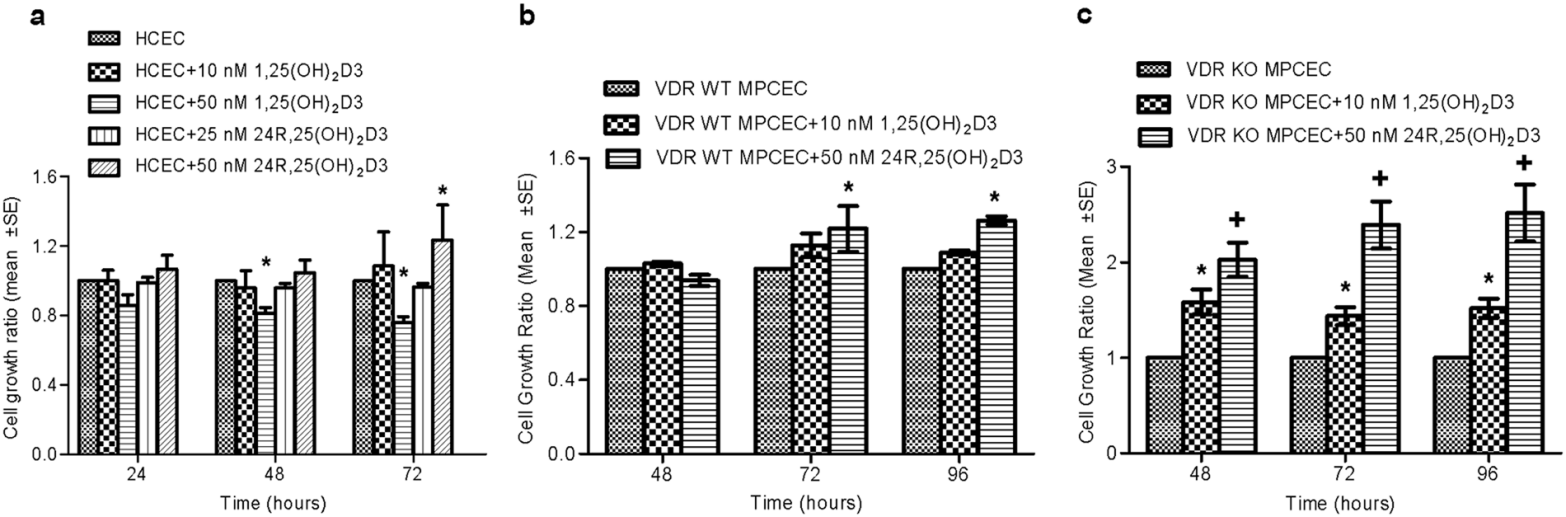
Effects of Vit D metabolites on human and mouse cell proliferation as measured with the MTT assay. (**a**) 24R,25(OH)_2_D3 (50 nM) significantly increased HCEC (*P < 0.05, n = 5), (**b**) VDR WT MPCEC (*P < 0.05, n = 5), and (**c**) VDR KO MPCEC proliferation (^+^P < 0.01, n = 5). There was no significant effect of 10 nM 1,25(OH)_2_D3 on HCEC or VDR WT MPCEC proliferation. 1,25(OH)_2_D3 (50 nM) significantly inhibited HCEC proliferation (*P < 0.05, n = 5) (**a**). 10 nM 1,25(OH)_2_D3 significantly increased VDR KO MPCEC proliferation (*P < 0.05, n = 5) (**c**).

In HCEC and MPCEC cultured from VDR wildtype mice (VDR WT MPCEC), 100 nM 24R,25(OH)_2_D3 significantly increased cell proliferation at the 72 and 96 hour time points, respectively, while 25 nM 24R,25(OH)_2_D3 had no effect (Fig. 2a,b). 10 nM 1,25(OH)_2_D3 had no effect on these cells, while 50 nM 1,25(OH)_2_D3 significantly decreased HCEC proliferation at 48 and 72 hours. In mouse primary corneal epithelial cells cultured from VDR knockout mice (VDR KO MPCEC), both 10 nM 1,25(OH)_2_D3 and 100 nM 24R,25(OH)_2_D3 significantly increased cell proliferation at the 48, 72 and 96 hours time points (Fig. 2c).

### Expression of CYP24A1, CYP27B1

**Figure 3:**
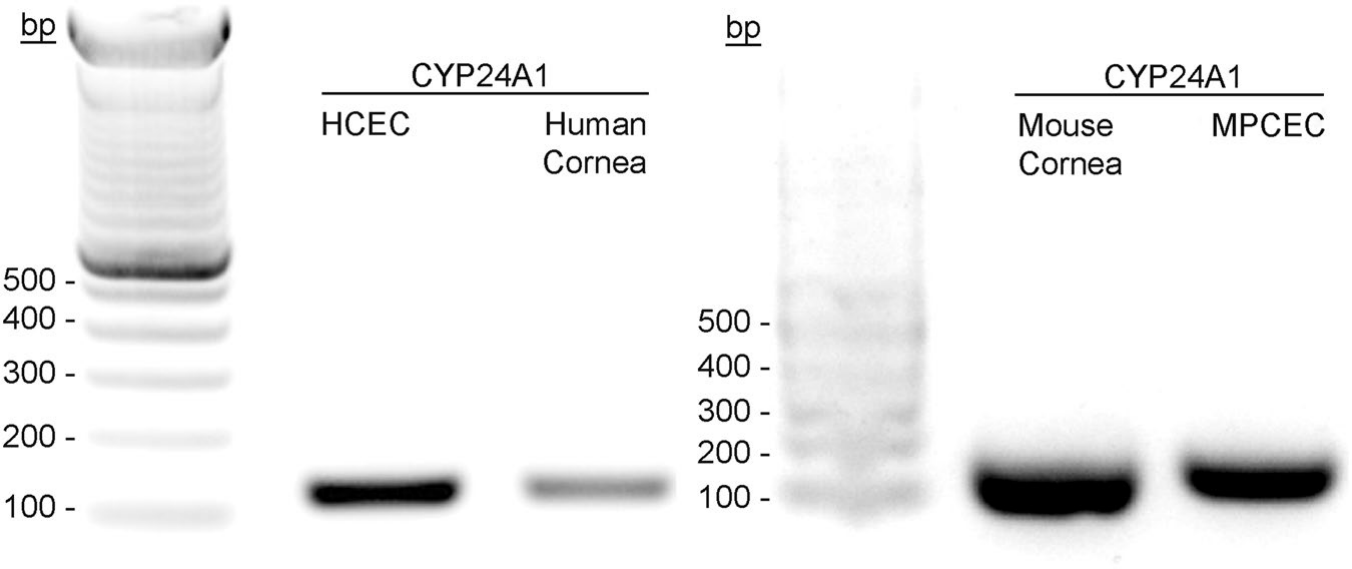
CYP24A1 mRNA was detected in HCEC, human corneal tissue (three human donors), fresh mouse corneal epithelial cells (three mice), and VDR WT MPCEC using the reverse transcription polymerase chain reaction in 2% Agarose gel. Fresh tissue data is representaive from 3 human corneas and three mice (6 corneas).

**Figure 4:**
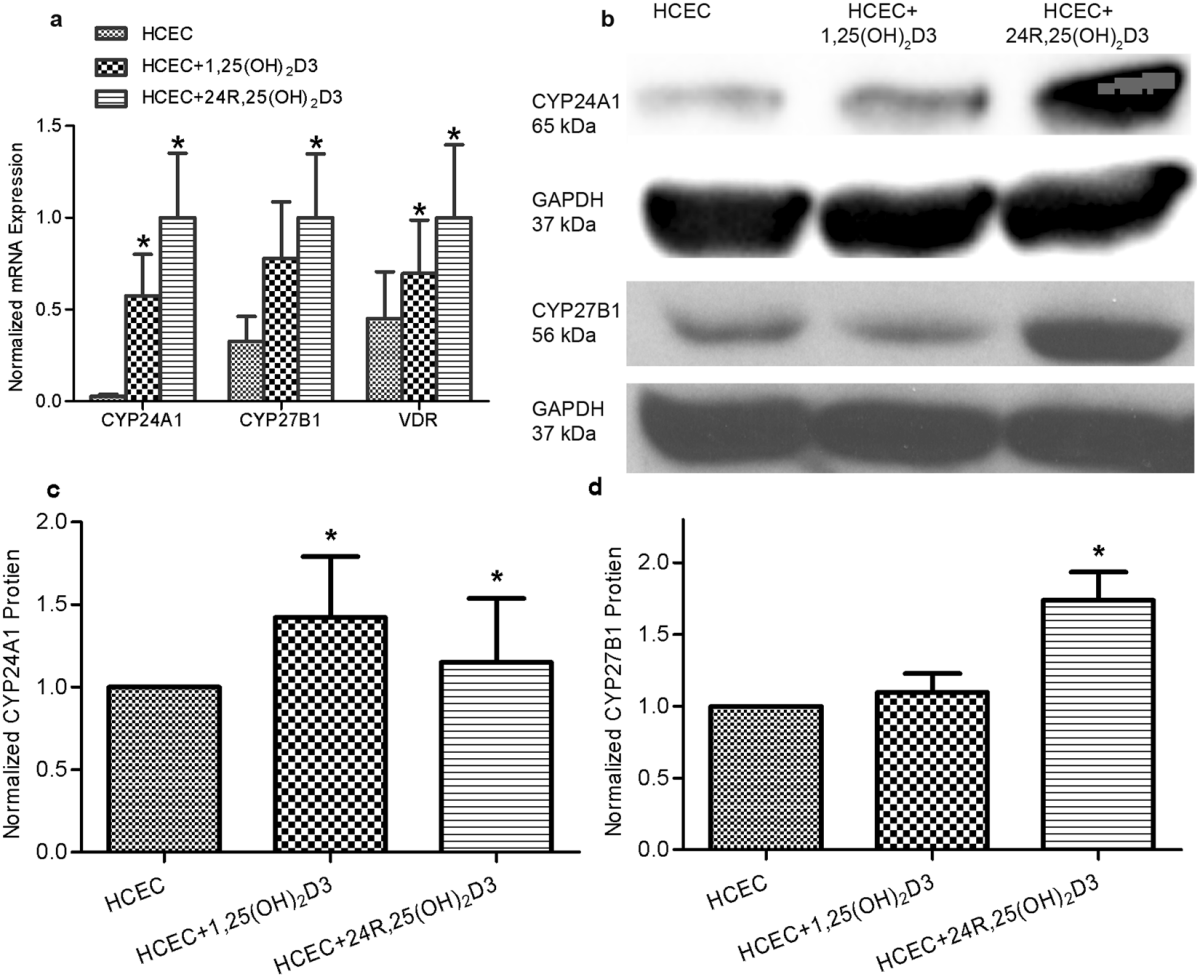
(**a**) CYP24A1, CYP27B1, and VDR mRNA levels were significantly increased in HCEC treated with 1,25(OH)_2_D3 or 24R,25(OH)_2_D3 (*P < 0.01, n = 3). The melt curves shown in Supplemental figures S2a,b show the RT-PCR products detected in in 2% Agarose gel. (**b**,**c**) CYP24A1 protein expression was significantly increased in HCEC treated with both 1,25(OH)_2_D3 and 24R,25(OH)_2_D3 (*P < 0.05, n = 3, ChemiDoc™ XRS^+^ Imaging System) while CYP27B1 protein expression was significantly increased only in HCEC treated with 24R,25(OH)_2_D3 (*P < 0.05, n = 3, X-ray film imaging).

**Figure 5:**
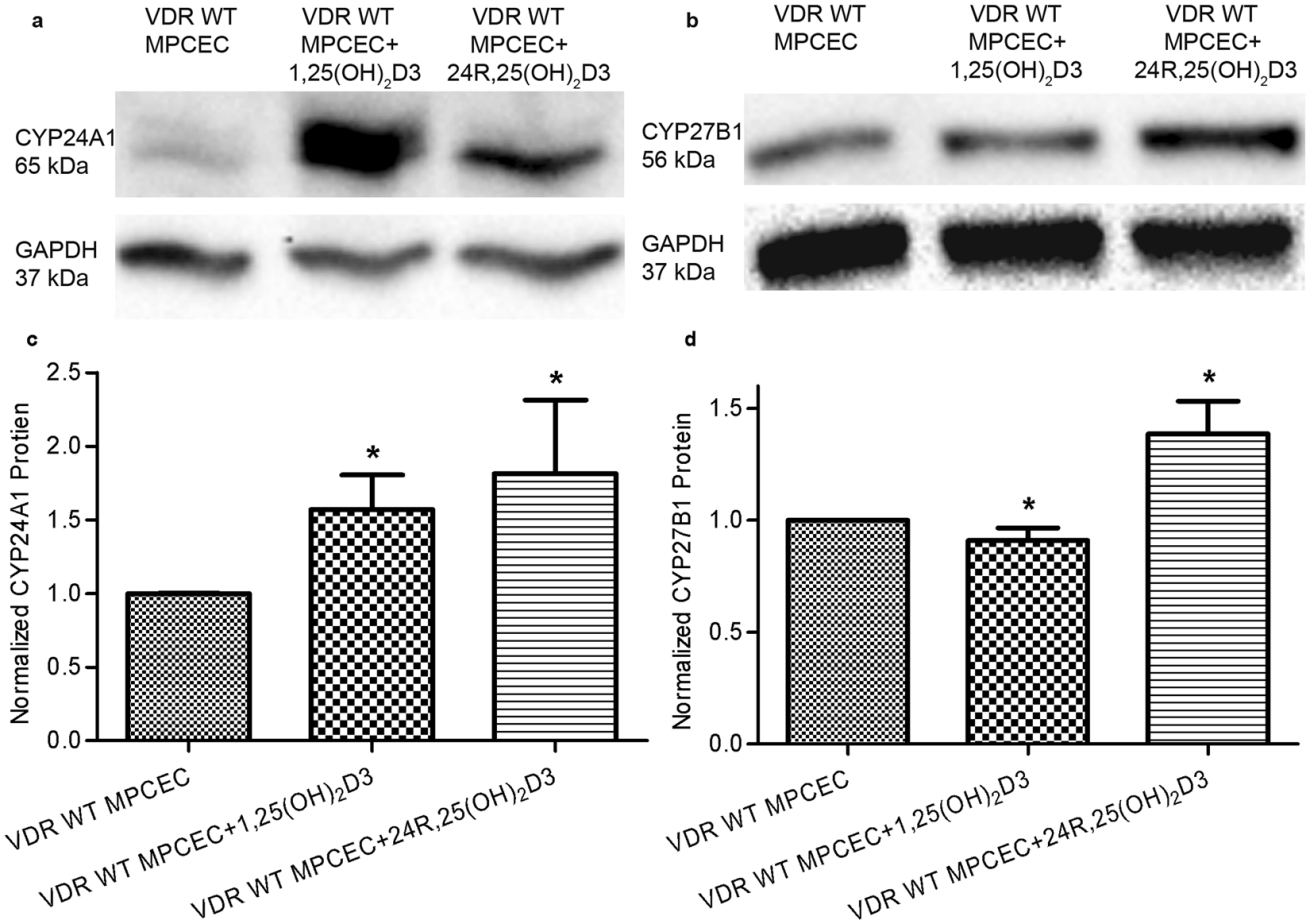
CYP24A1 protein expression was significantly increased in VDR WT MPCEC treated with 1,25(OH)_2_D3 and 24R,25(OH)_2_D3 (*P < 0.05, n = 3, ChemiDoc™ XRS^+^ Imaging System). CYP27B1 protein was significantly increased in VDR WT MPCEC treated with 24R,25(OH)_2_D3 (*P < 0.05, n = 3), and significantly decreased in VDR WT MPCEC treated with 1,25(OH)_2_D3 (*P < 0.05, n = 3).

PCR results were positive for CYP24A1 mRNA in all cells and tissue examined, including fresh human corneal epithelium pooled from corneal tissue, in the HCEC cell line, and in fresh mouse corneal epithelium pooled from corneal tissue, and cultured primary mouse corneal epithelium (Fig. 3). Both 1,25(OH)_2_D3 and 24R,25(OH)_2_D3 significantly induced CYP24A1 mRNA (Fig. 4a) and protein expression (Fig. 4b,c) in HCEC. 24R,25(OH)_2_D3 significantly induced mRNA and protein expression of CYP27B1 in HCEC. There was no significant change in protein expression of CYP27B1 in HCEC treated with 1,25(OH)_2_D3, although the mRNA expression was significantly increased. Protein expression of both CYP24A1 and CYP27B1 in VDR WT MPCEC increased following treatment with 24R,25(OH)_2_D3 (Fig. 5). 1,25(OH)_2_D3 significantly increased protein expression of CYP24A1 and reduced protein expression of CYP27B1 in VDR WT MPCEC.

### Effects of VDR silencing

**Figure 6:**
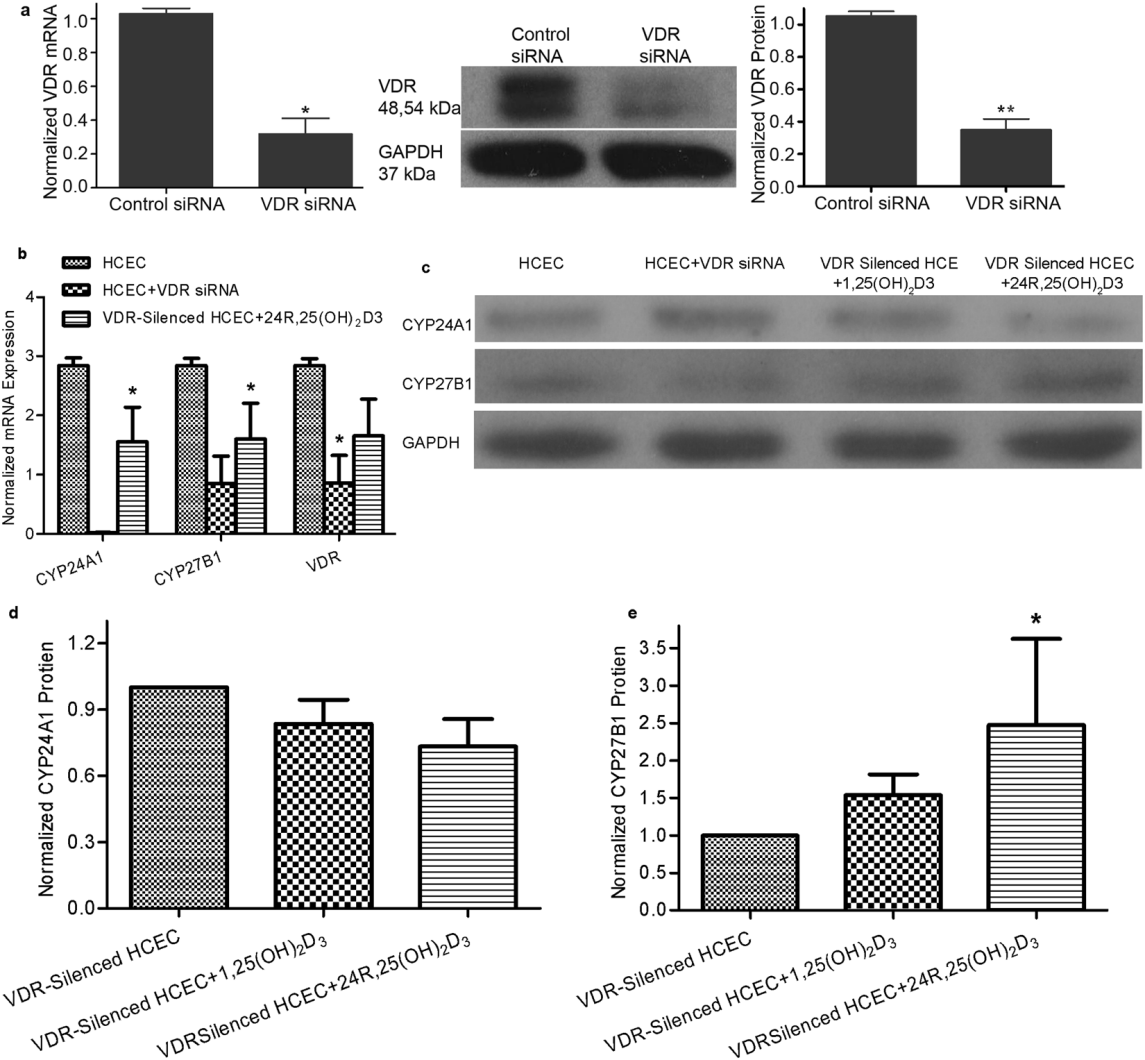
HCEC cultured with VDR siRNA for 48 h and treated with 1,25(OH) _2_D3 and 24R,25(OH)_2_D3 for 16 h. (**a**) siRNA treatment resulted in a greater than 70% decrease in VDR mRNA (left graph), and VDR protein level (*P < 0.05, **P < 0.01, n = 3) (gel and right graph). (**b**) VDR silencing resulted in significantly lower CYP24A1 and CYP27B1 mRNA expression, while in these VDR silenced cells, 24,25-Vitamin D3 treatment resulted in significantly increased expression of both CYP24A1 and CYP27B1 mRNA levels (*P < 0.01, n = 3) compared to the untreated VDR knockdown levels. Melt curves are shown in Supplemental Figure S3. (**c**,**d**) CYP27B1 protein expression was increased in VDR-silenced HCEC cultured with 24R,25(OH)_2_D3, while CYP24A1 protein expression was not affected by either 1,25(OH) _2_D3 or 24R,25(OH)_2_D3 in VDR-silenced HCEC (*P < 0.05, n = 3, X-ray film imaging).

Silencing and knockout of VDR gene expression was used to examine whether 1,25(OH)_2_D3 and 24R,25(OH)_2_D3 can act on HCEC independently of the VDR pathway. Treatment with VDR siRNA resulted in a more than 70% reduction of VDR mRNA expression compared to control cells (Fig. 6a). CYP24A1 and CYP27B1 mRNA were significantly decreased in VDR-silenced HCEC. 24R,25(OH)_2_D3 increased CYP24A1 and CYP27B1 mRNA expression in VDR silenced HCEC (Fig. 6b) compared to the untreated VDR knockdown levels. CYP27B1 protein expression was significantly increased in VDR silenced HCEC cultured with 24R,25(OH)_2_D3 but not with 1,25(OH)_2_D3 (Fig. 6c,e). CYP24A1 protein expression was not significantly changed in VDR silenced HCEC cultured with 1,25(OH)_2_D3 or 24R,25(OH)_2_D3 (Fig. 6c,d).

### Effects of VDR knockout

**Figure 7:**
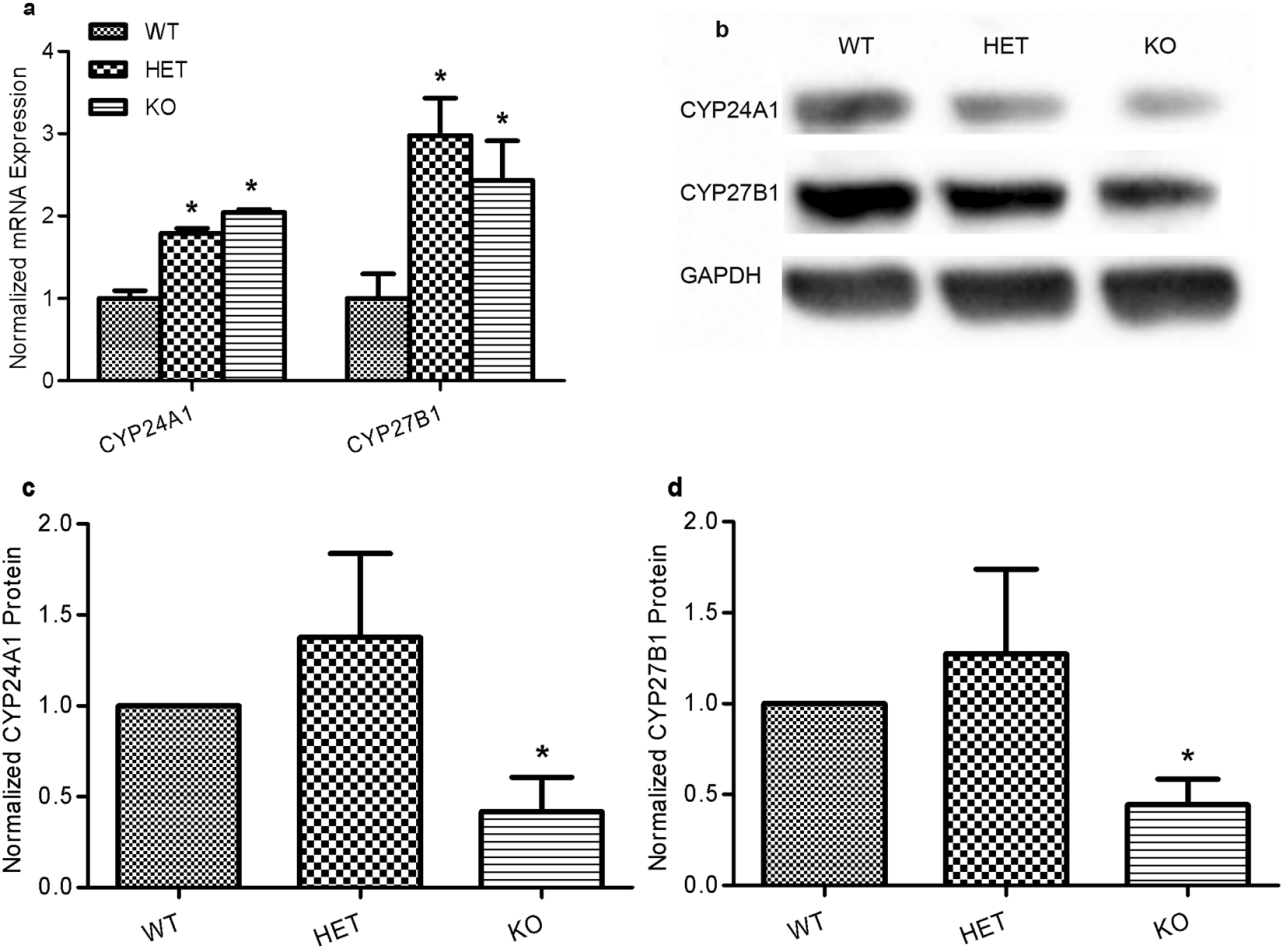
CYP24A1 and CYP27B1 measured in fresh (not cultured) epithelium from VDR WT, HET and KO mice. (**a**) CYP24A1 and CYP27B1 mRNA expression was increased in epithelium from VDR HET and KO mice compared to VDR WT mice (*P < 0.05, n = 3). The melt curves shown in Supplemental Figure S4 show the RT-PCR product detected in in 2% Agarose gel. (**b**,**c**) CYP24A1 and CYP27B1 protein expression was unaffected in HET mouse epithelial cells and was decreased in VDR KO epithelium compared to VDR WT mice (*P < 0.05, n = 3. ChemiDoc™ XRS^+^ Imaging System).

**Figure 8:**
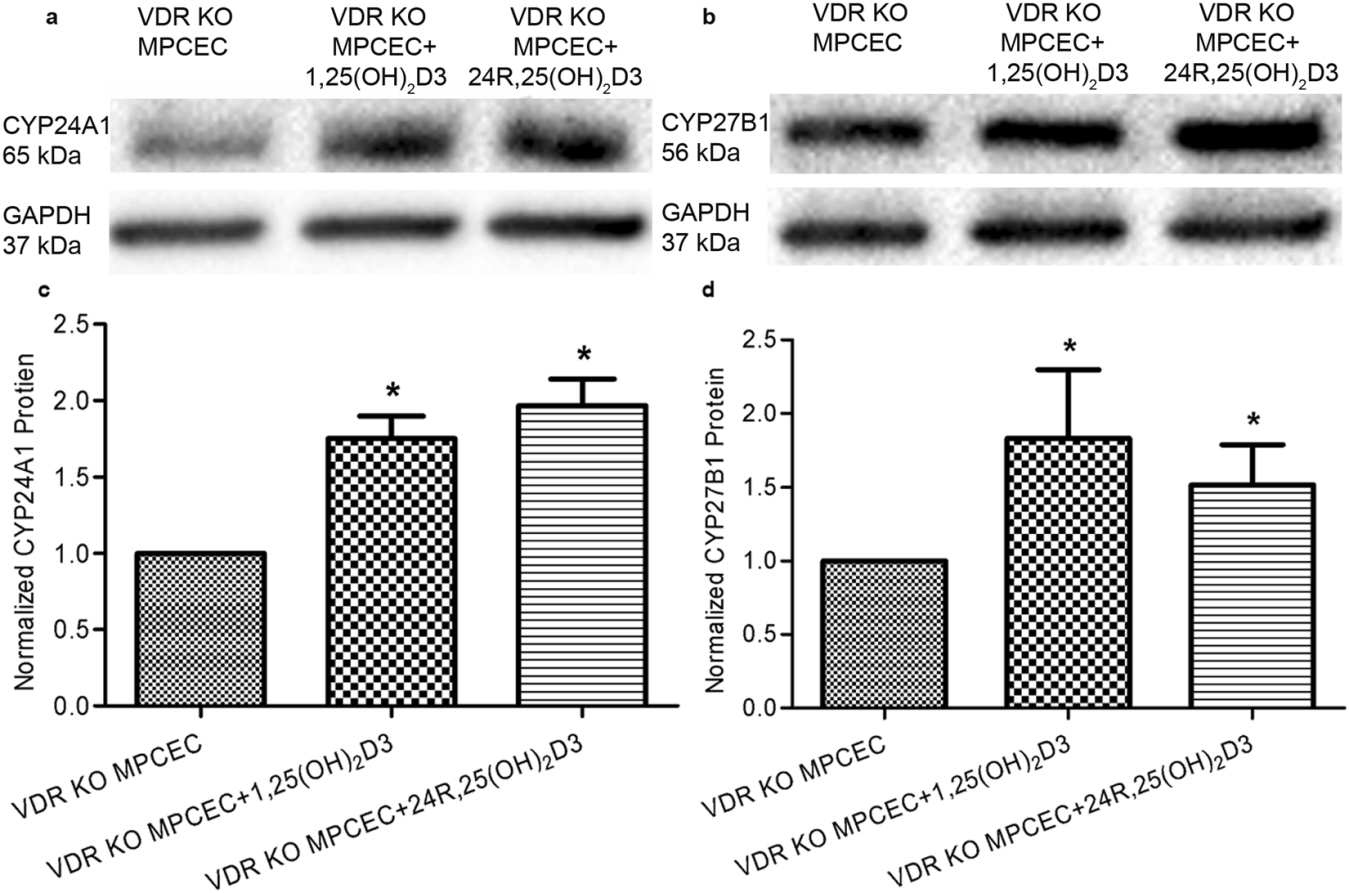
VDR KO MPCEC treated with 1,25(OH) _2_D3 and 24R,25(OH)_2_D3. CYP24A1 and CYP27B1 protein levels were both significantly increased in VDR KO MPCEC treated with 1,25(OH)_2_D3 and 24R,25(OH)_2_D3 (*P < 0.01, n = 3, ChemiDoc™ XRS^+^ Imaging System).

Compared to wild type mice, there was a significiant increase in CYP24A1 and CYP27B1 mRNA expression in fresh corneal epithelial cells pooled from VDR HET and KO mice (Fig. 7a). However, CYP24A1 and CYP27B1 protein expression was significantly lower in VDR KO mice (Fig. 7b,c). Protein levels were unaffected in cells from HET mice. To further explore the role of VDR in the vitamin D enzyme feedback loop, primary corneal epithelial cells from VDR knockout mice were cultured and exposed to 1,25(OH)_2_D3 and 24R,25(OH)_2_D3. Both 1,25(OH)_2_D3 and 24R,25(OH)_2_D3 treatment resulted in significantly increased CYP24A1 and CYP27B1 protein expression (Fig. 8).

### Original Scans

Original scans of all western blots are shown in Supplemental Figures S5–S9.

## Discussion

The corneal epithelium has unique structural and physiologic properties that allow it to fulfill its functions in the eye. Using mass spectroscopy, our group previously examined vitamin D metabolites present in the ocular fluids of New Zealand white rabbits and also examined the influence of dietary vitamin D supplementation on these fluids. We detected relatively high concentrations of the metabolite 1,25(OH)_2_D2 in control rabbits compared to low or minimal concentrations of the other vitamin D metabolites including 25(OH)D3, 1,25(OH)_2_D3 and 24R,25(OH)_2_D3^22,33^. Tear and aqueous humor 25(OH)D3 and 24,25(OH)_2_D3 levels were significantly elevated following Vit D supplementation. Furthermore, our lab previously reported vitamin D enhancement of tight junction resistance and gap junction cell to cell diffusion coefficients^7^. We also demonstrated attenuated corneal epithelial wound healing in 10-week-old VDR knockout mice^34^, which is similar to the effects of deletion of VDR deletion in mouse skin which resulted in retarding wound healing^35,36^. Deletion of VDR has also been shown to reduce β-catenin transcriptional activity and proliferation of cells at the leading edge of wound closure, and vitamin D was found to be required for a normal regenerative response of the skin to wounding^37,38^. Vitamin D has been found to have anti-inflammatory and immunoregulatory roles, and deficiency could result in dry eye disease^39,40^. The relationship of vitamin D to other ocular pathologies has also been studied, and the therapeutic potential of vitamin D has been examined^41^. In our current study, both 1,25(OH)_2_D3 and 24R,25(OH)_2_D3 significantly increased primary human epithelial cell migration. Therefore, anterior segment hypovitaminosis D could be detrimental after corneal injury, where vitamin D3 appears to play a role in the cell processes that occur during the corneal epithelial wound healing process as the cells migrate to cover the wound and differentiate into a multi-layered, mature corneal epithelium.

Vitamin D has been shown to influence cell differentiation and proliferation^6,42^. Most of the literature describes an inhibitory effect of 1α,25(OH)_2_D3 on cell proliferation in various cell types, often associated with stimulation of cell differentiation. Our current study found that while both 1α,25(OH)_2_D3 and 24,25(OH)_2_D3 stimulated migration, 50 nM 1,25(OH)2D3 inhibited HCEC proliferation (Fig. 1A). In agreement with this, Reins *et al*. recently found a small but significant inhibitory effect of topically applied 100 nM 1α,25(OH)_2_D3 on mouse corneal epithelial wound healing^43^. It is possible that while proliferation is inhibited, differentiation may be stimulated. Interestingly, we found that both 1α,25(OH)_2_D3 and 24,25(OH)_2_D3 significantly increase epithelial proliferation in VDR KO MPCEC. Taken together with the data in Fig. 2 showing that 1α,25(OH)_2_D3 inhibits proliferation in HCEC, the data indicates that that VDR stimulation is likely inhibitory, while 1α,25(OH)_2_D3 and 24,25(OH)_2_D3 can act through a non-VDR mediated pathway to stimualte proliferation.

It is generally believed that 24,25(OH)_2_D3 is an inactive form of vitamin D3. This belief has been changing as of late, in that 24,25(OH)_2_D3 has been shown to effect bone mass and the differentiation and maturation of growth plate chondrocytes *in vitro* ^44–46^. Moreover, a recent study of multiple sclerosis patients demonstrated that the ratio of serum 25(OH)D3 to 24R,25(OH)_2_D3 and not the ratio of 25(OH)D3 to 1,25(OH)2D3 correlated with higher disability and increased disease progression and brain atrophy^47^. In addition, 24R,25(OH)_2_D3 has been shown to increase bone healing and fracture healing^23^. 24R,25(OH)_2_D3 was also demonstrated to be important during human mesenchymal stem cell maturation, and has an essential role in Ca^2+^ mineralization, gene expression, and the regulation of cytochrome P450 expression^24^. There have been no previous studies examining the possible function(s) of 24R,25(OH)_2_D3 in the cornea. The current study demonstrates that 24R,25(OH)_2_D3 stimulates both HCEC cell proliferation and migration. As both of these actions are crucial for corneal epithelial wound healing, these results provide evidence that 24R,25(OH)_2_D3 is likely involved in, and beneficial for corneal wound healing, as it is in bone and fracture healing.

CYP27B1 is the only enzyme known to catalyze 1α-hydroxylation of 25(OH)D3, and CYP24A1 is the only known enzyme capable of catalyzing 24-hydroxylation of 25(OH)D^48^. The action of CYP24A1 on 25-hydroxyvitamin D results in decreased 1,25(OH)_2_D3 levels and increased 24R,25(OH)_2_D3, while CYP27B1 increases 1,25(OH)_2_D3 levels^49^. In our studies, the CYP24A1 gene and protein expression level are highly inducible by 1,25(OH)_2_D3 in HCEC and primary mouse corneal primary epithelial cells, as in several other tissues, thus acting as a local control mechanism to prevent tissue-level 1,25(OH)_2_D3 intoxication^44,50^. In addition, our results indicate that stimulation of CYP24A1and the resultant increase in 24R,25(OH)_2_D3 levels will likely lead to stimulation of epithelial cell migration and proliferation as described above. Reins *et al*. recently described that the expression of CYP24A1 mRNA in HCEC and human primary corneal epithelial cells was increased with 1,25(OH)_2_D3 or 25(OH)D3 through a functional VDR, as silencing of the receptor blocked this response^9^. While we did not examine the influence of 1,25(OH)_2_D3 or 25(OH)D3 on CYP24A1 mRNA, we did find that CYP24A1 and CYP27B1 mRNA and protein were increased in VDR-silenced HCEC cultured with 24R,25(OH)_2_D3. Interestingly, CYP24A1 protein expression was not affected by 1,25(OH)_2_D3 or 24R,25(OH)_2_D3 in VDR-silenced HCEC. This would be a not so uncommon example of protein expression not mimicking mRNA expression. Previous studies have demonstrated that while 1,25(OH)_2_D3-dependent regulation of DNA synthesis in cartilage cells requires VDR^18,51,52^, other physiological responses to 1,25(OH)_2_D3 involve regulation via the Pdia3 membrane receptor (also called protein disulfide isomerase, ERp57,GR58, and 1,25D3-MARRS)^53–55^.

The current study also used cells obtained and cultured from VDR KO mice to further explore the role of VDR in corneal epithelial cell function. Unlike the VDR silencing studies, 1,25(OH)_2_D3 and 24R,25(OH)_2_D3 were both found to promote CYP24A1 and CYP27B1 protein expression in VDR KO MPCEC. The differing protein CYP24A1 and CYP27B1 expression levels in VDR silencing versus knockout studies may be a species specific result or a side-effect of either the gene knockout or silencing procedures. The differences are unlikely due to incomplete silencing of VDR in the siRNA study as gene silencing typically results in continued (although reduced) gene function in silenced cells versus total loss of function as in KO cells, which is not the response found in this study. Interestingly, significantly greater cell growth was observed in VDR KO mouse cells exposed to 1,25(OH)2D3 or 24R,25(OH)2D3 than was observed for WT mouse corneal epithelial cells (Fig. 2). This data indicates that there is a Vit D signaling pathway in corneal epithelium that is distinct from the traditional VDR pathway, and that VDR may be a negative regulator of corneal epithelial cell growth.

We report, for the first time, 24R,25(OH)2D3-initiated feedback control of the key vitamin D enzymes CYP24A1 and CYP27B1 through VDR-independent signaling in the corneal epithelium. A 24R,25(OH)_2_D_3_ positive feedback loop has been reported in osteoblasts, where 24R,25(OH)_2_D3 markedly enhanced the CYP24A1 mRNA level while not effecting CYP27B1 mRNA, which may result in a higher production of 24R,25(OH)_2_D_3_^25^. Our findings demonstrate that 24R,25(OH)_2_D3 significantly increases CYP24A1 and CYP27B1 expression in HCEC, VDR WT MPCEC, and VDR KO MPCEC. 1,25(OH)2D3 also had effects on CYP24A1 and CYP27B1 in VDR KO MPCEC. Additional functions of 24R,25(OH)_2_D3 in Vitamin D metabolism and catabolism need to be further exploreed in corneal epithelial cells.

In summary, CYP24A1 is expressed in human cornea, mouse cornea, and MPCEC. 1,25(OH)_2_D3 increases CYP24A1 mRNA and protein expression in HCEC, VDR WT MPCEC, and VDR KO MPCEC. HCEC proliferation is significantly increased following treatment with 24R,25(OH)2D3, but not with 1,25(OH)2D3. 1,25(OH)_2_D3 and 24R,25(OH)_2_D3 both significantly increased primary human corneal epithelial cell migration. VDR WT MPCEC proliferation was also significantly increased following treatment with 24R,25(OH)2D3. Thus, we provide evidence that the 24-hydroxylated metabolite of vitamin D, 24R,25(OH)2D3, is not merely a degradation product, but instead exerts distinct effects on corneal epithelial cells. 24R,25(OH)2D3 increases expression of CYP27B1 in HCEC with or without VDR silencing, and in VDR WT and KO MPCEC, indicating the involvement of a non-VDR 24R,25(OH)2D3 signaling pathway.

## Electronic supplementary material

Supplementary Information
