# Supplementary material for: Effects of 1,25 and 24,25 Vitamin D on Corneal Epithelial Proliferation, Migration and Vitamin D Metabolizing and Catabolizing Enzymes
Source: Sci Rep. 2017 Dec 5;7:16951. doi: 10.1038/s41598-017-16698-3 (PMC5717139; doi:10.1038/s41598-017-16698-3)

**Supplementary Data**

**Effects of 1,25 and 24,25 Vitamin D on Corneal Epithelial Proliferation, Migration and Vitamin D Metabolizing and Catabolizing Enzymes**

Xiaowen Lu^1^, Zhong Chen^1^, Namratha, Mylarapu^1^, Mitchell A. Watsky^1,2*^

^1^Department of Cellular Biology and Anatomy, Medical College of Georgia, Augusta University, Augusta, GA

^2^The Graduate School, Augusta University, Augusta, GA

**Supplemental Table S1**

**Table S1 Efficiency of CYP24A1, CYP27B1, GAPDH and TBP primers.**

| Primer Efficiency^a^  Gene | Human | Mouse |
| --- | --- | --- |
| CYP24A1 | 108% | 100% |
| CYP27B1 | 93% | 90% |
| GAPDH | 105% |  |
| TBP |  | 106% |

^a^Determination of primer efficiency using the Cq slope method. PCR template was the PCR product of purified 1 μg mRNA from HCEC with Bio-Rad iScript^TM^ cDNA Synthesis Kit for Human CYP24A1, CYP27B1 and GAPDH, and 1 μg mRNA from mouse kidney was used for mouse CYP24A1, CYP27B1 and TBP.

**Supplemental Figure S1**

The cornea epithelium specific markers keratin 12 (K12) and mucin 1 in HCEC and primary human corneal epithelial cells (HPCEC) were detected by reverse transcription polymerase chain reaction in 2% Agarose gel.


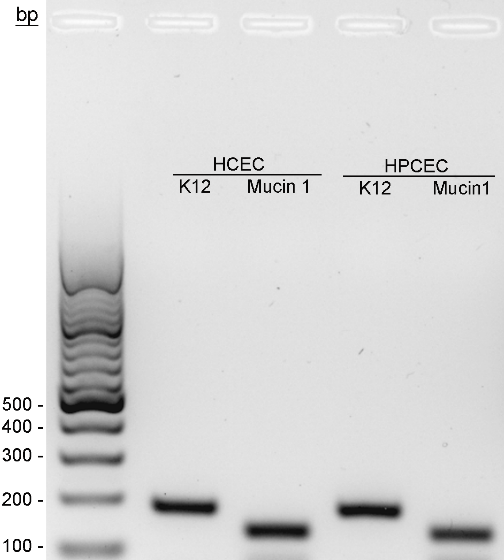


**Supplemental Figure S2**

The mRNA of VDR, CYP24A1 and CYP27B1 were detected in human corneal epithelial cells using the reverse transcription polymerase chain reaction in 2% Agarose gel with designed primers which yielded specific products (Figure 3a). The melt curves (Figure 3b) are shown for the expression of CYP24A1, CYP27B1 and the reference gene GAPDH from HCEC cultured with 1,25(OH)_2_D3 and 24R,25(OH)_2_D3. Horizontal lines represent threshold values for the indicated targets.

Figure S2a


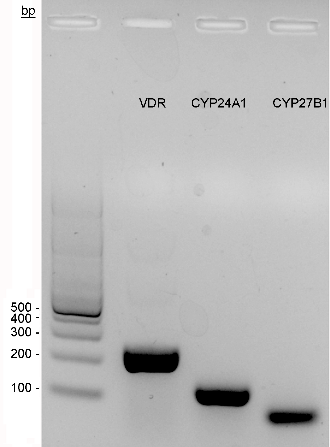


Figure S2b

**
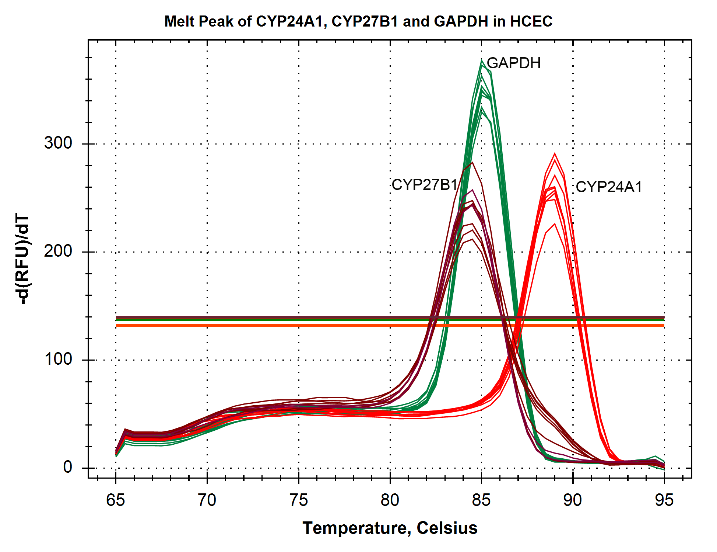
**

**Supplemental figure S3**

The melt curves for the expression of CYP24A1, CYP27B1, VDR and the reference gene GAPDH in VDR-silenced HCEC cultured with 24R,25(OH)_2_D3. Horizontal lines represent threshold values for the indicated targets.

**
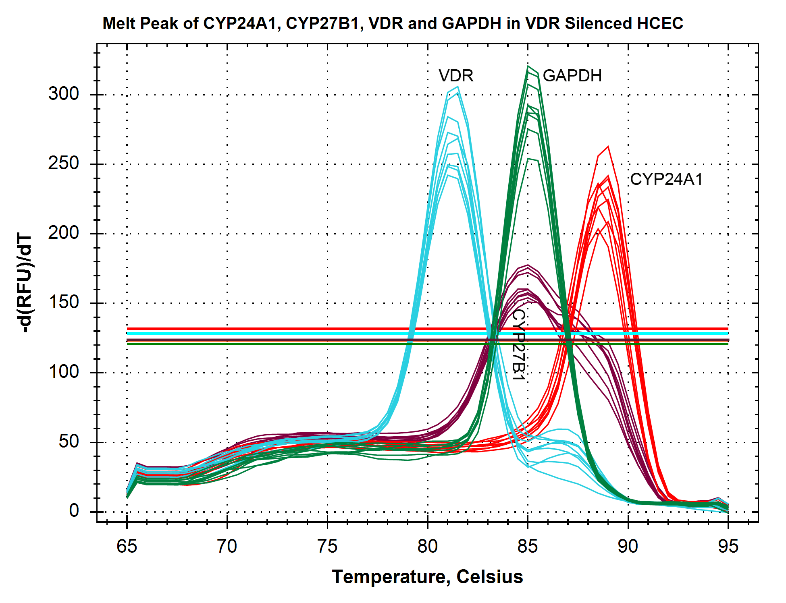
**

**Supplemental figure S4**

The mRNA of CYP24A1 and CYP27B1 were detected in wildtype mouse corneal epithelial cells using reverse transcription polymerase chain reaction in 2% Agarose gel with designed primers which yielded specific products (Figure 4a). The melt curves (Figure 4b) for the expression of CYP24A1, CYP27B1 and the reference gene TBP in corneal epithelial cells from tissue of VDR knockout homozygous, heterozygous and wildtype mice. Horizontal lines represent threshold values for the indicated targets.

Figure S4a


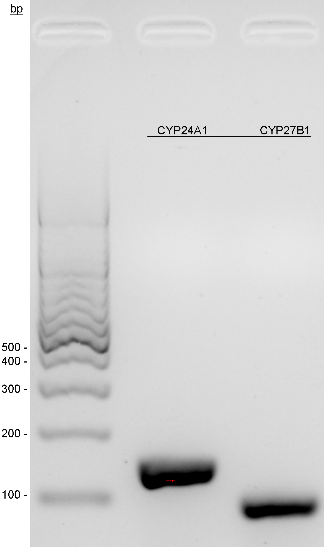


Figure S4b


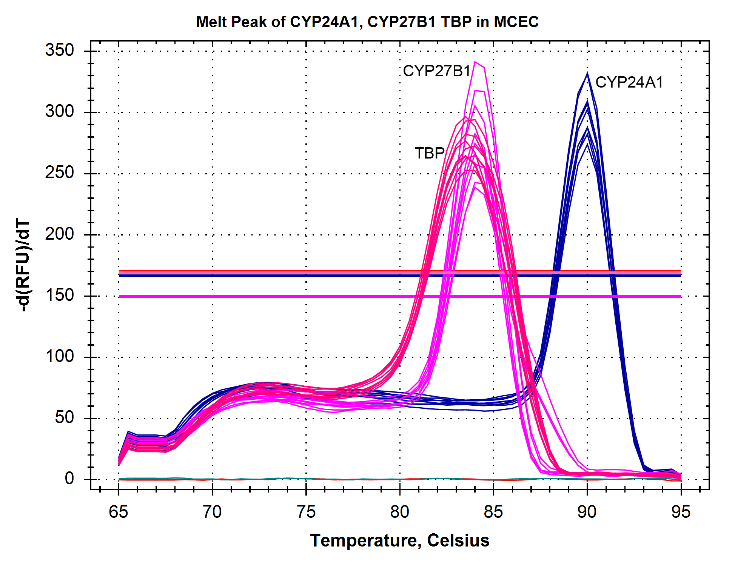


**Supplemental figure S5**

Original scans of blot for Figure 4b. CYP24A1 protein expression was significantly increased in HCEC treated with both 1,25(OH)_2_D3 and 24R,25(OH)_2_D3 (*P<0.05, n=3, ChemiDoc™ XRS^+^ Imaging System). CYP27B1 protein expression (upper row) was significantly increased only in HCEC treated with 24R,25(OH)_2_D3 (*P<0.05, n=3, X-ray film imaging).


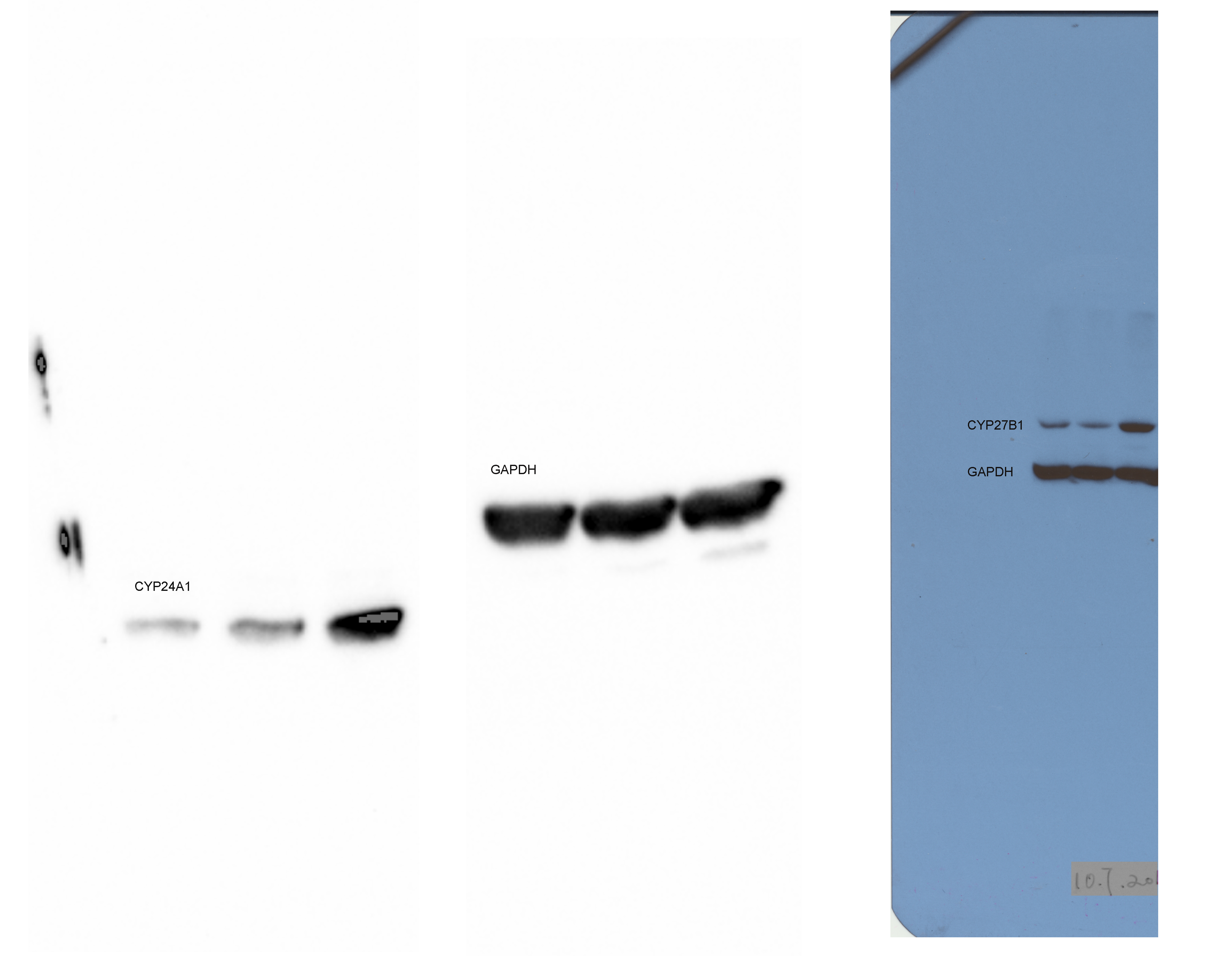


**Supplemental figure S6**

Original scans of blot for Figure 5a&b. CYP24A1 protein expression was significantly increased in VDR WT MPCEC treated with 1,25(OH)2D3 and 24R,25(OH)2D3 (*P<0.05, n=3). CYP27B1 protein was significantly increased in VDR WT MPCEC treated with 24R,25(OH)2D3 (*P<0.05, n=3), and significantly decreased in VDR WT MPCEC treated with 1,25(OH)2D3 (*P<0.05, n=3, ChemiDoc™ XRS+ Imaging System).


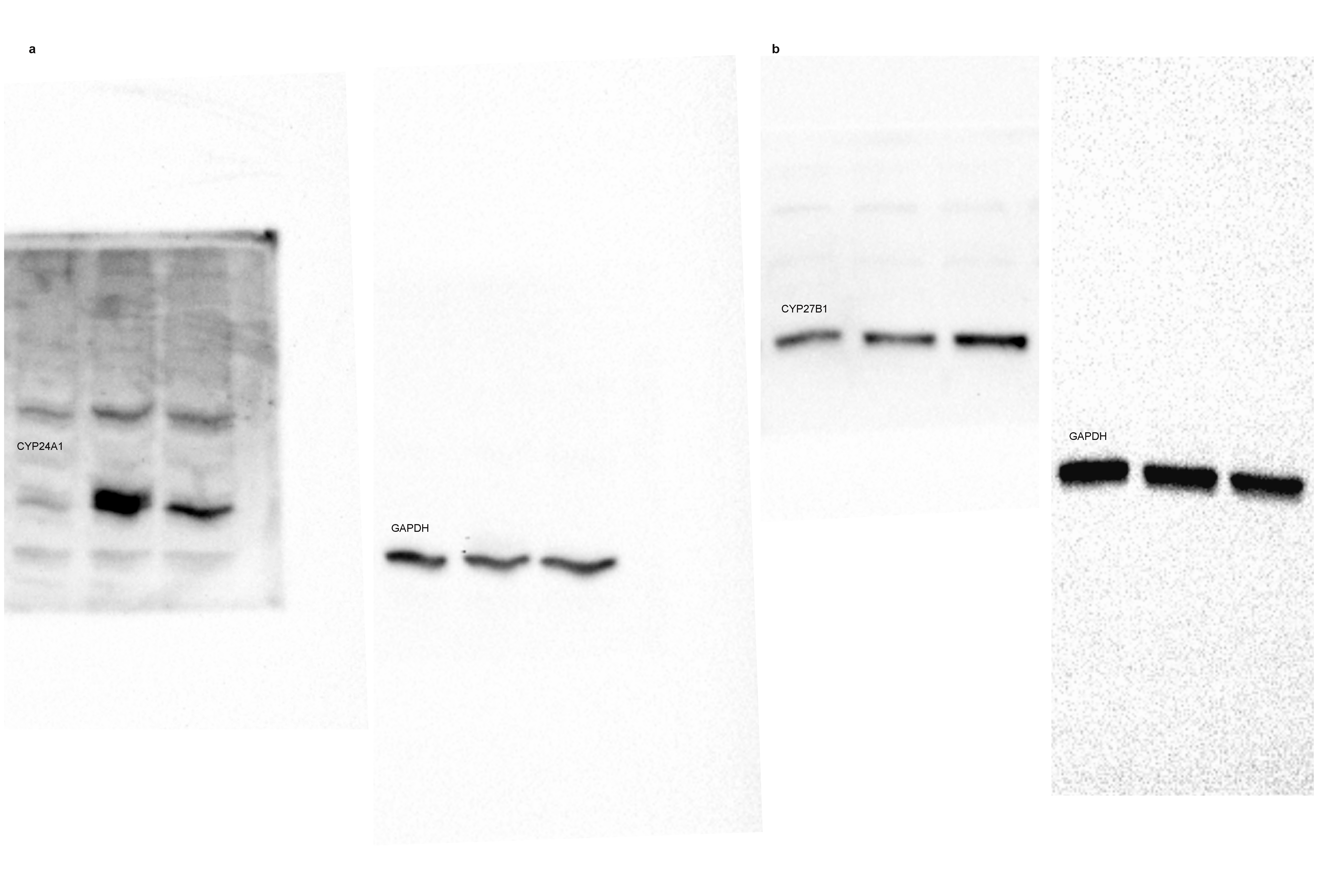


**Supplemental figure S7**

Original scans of blot for Figure 6a&c. VDR protein expression was detected in VDR-silenced HCEC. CYP27B1 protein expression was increased in VDR-silenced HCEC cultured with 24R,25(OH)_2_D3, while CYP24A1 protein expression was not affected by either 1,25(OH) _2_D3 or 24R,25(OH)_2_D3 in VDR-silenced HCEC (*P<0.05, n=3, X-ray film imaging).


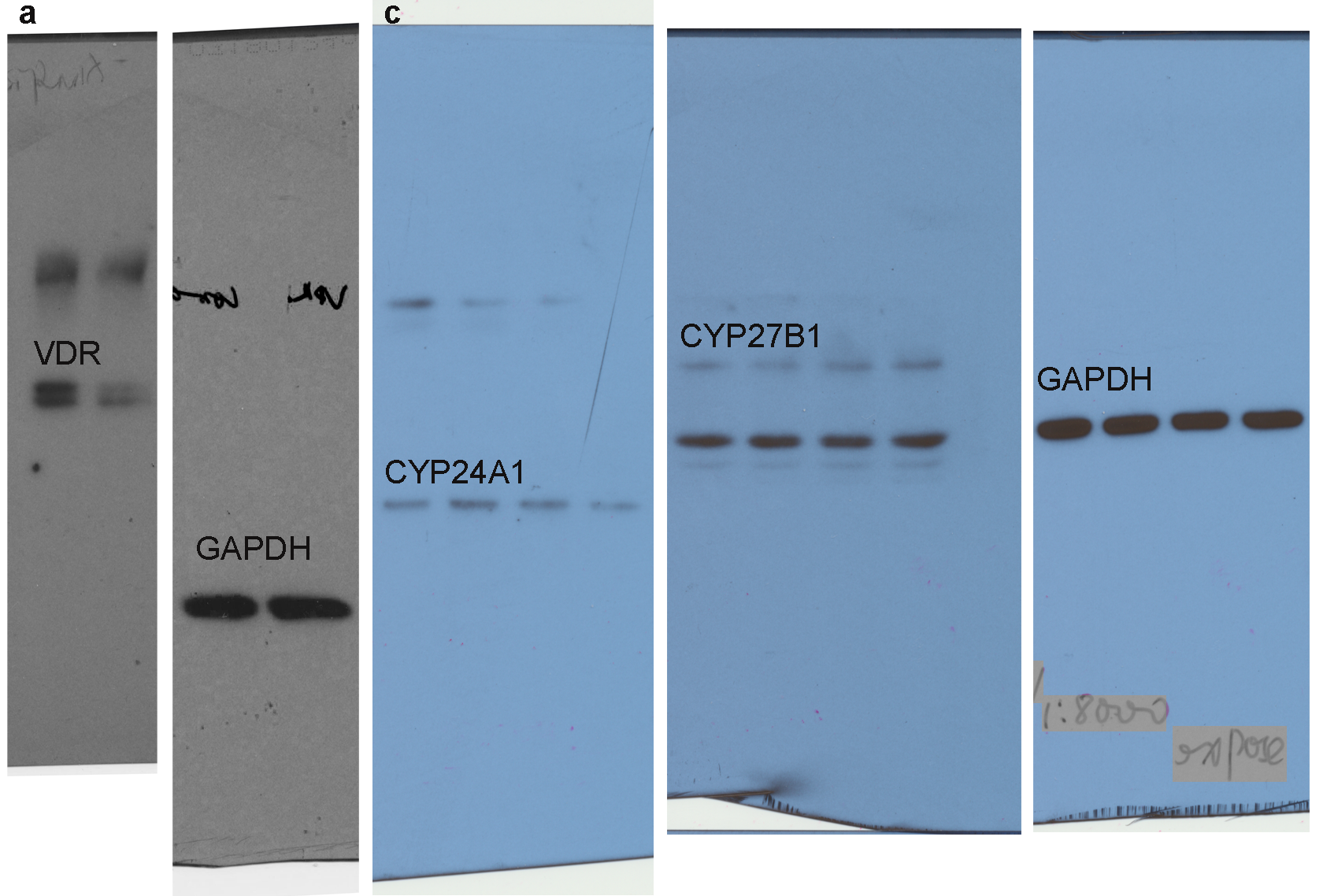


**Supplemental figure S8**

Original scans of blot for Figure 7b. CYP24A1 and CYP27B1 protein expression was unaffected in HET mouse epithelial cells and was decreased in VDR KO epithelium compared to VDR WT mice (*P<0.05, n=3. ChemiDoc™ XRS^+^ Imaging System).


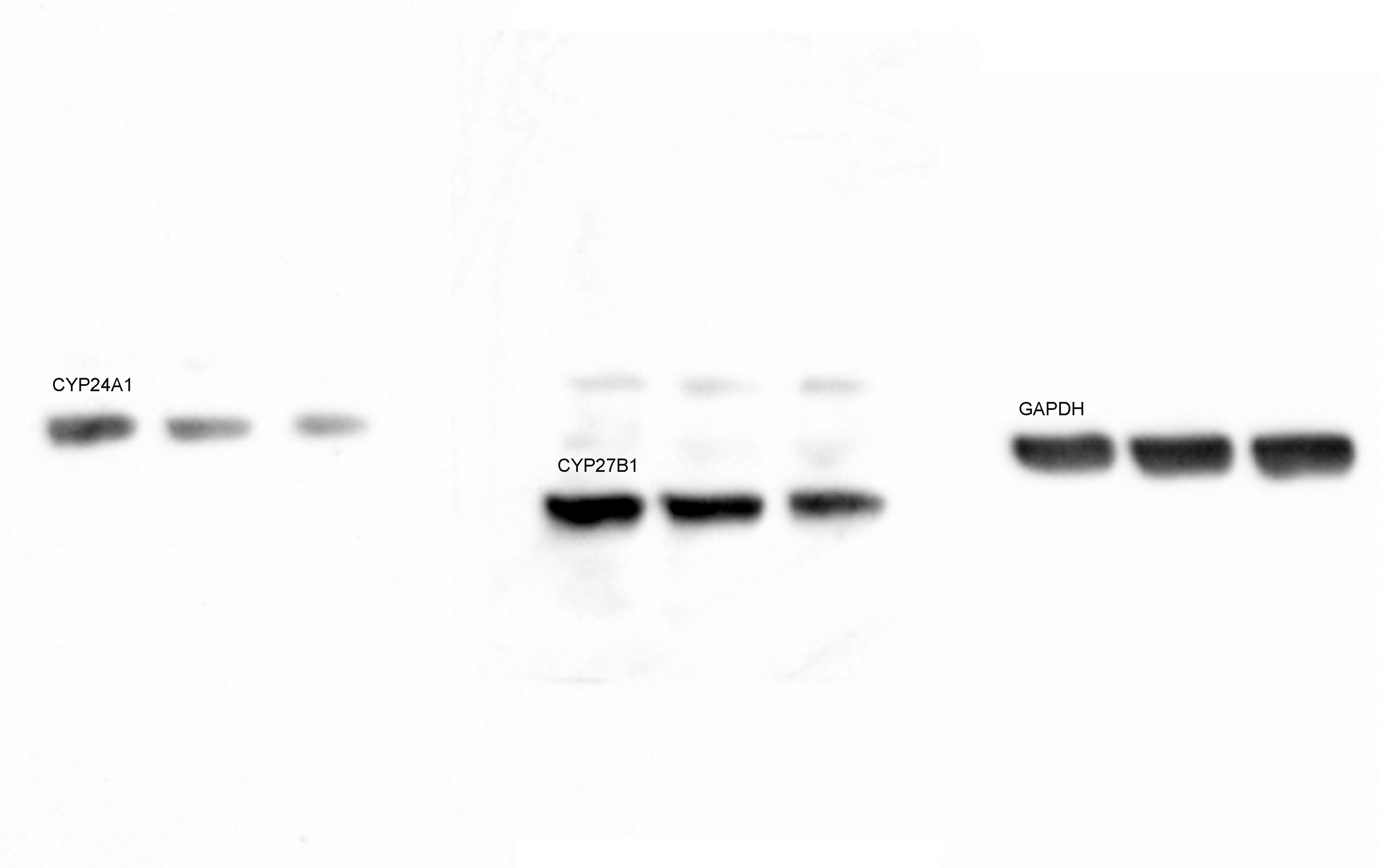


**Supplemental figure S9**

Original scans of blot for Figure 8a&b. CYP24A1 protein level was significantly increased in VDR KO MPCEC treated with 1,25(OH)2D3 and 24R,25(OH)2D3. CYP27B1 protein level was also significantly increased in VDR KO MPCEC treated with 1,25(OH)2D3 and 24R,25(OH)2D3 (*P<0.01, n=3, ChemiDoc™ XRS+ Imaging System).


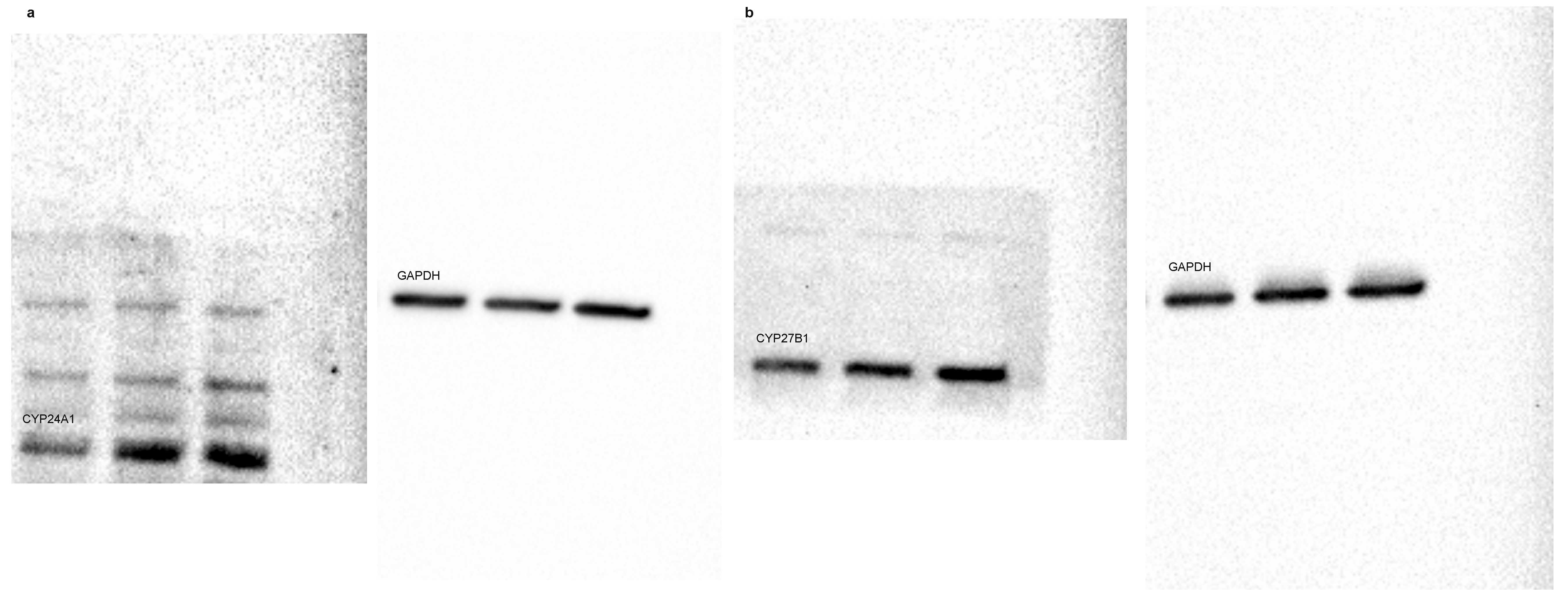

Supplement: Supplementary file 1 — Supplementary Information [file 41598_2017_16698_MOESM1_ESM.doc]
